# Neural Circuit Mechanisms of Disorders of Consciousness and Potential Therapeutic Targets of Brain Stimulation

**DOI:** 10.1002/cns.71037

**Published:** 2026-09-06

**Authors:** Yichen Zhou, Tianqing Cao, Sipeng Zhu, Qiheng He, Yitong Jia, Xiaoke Chai, Nan Wang, Yi Yang

**Affiliations:** ^1^ Department of Neurosurgery, Beijing Tiantan Hospital Capital Medical University Beijing China; ^2^ Department of Neurosurgery Aviation General Hospital Beijing China; ^3^ China National Clinical Research Center for Neurological Diseases Beijing China; ^4^ Innovative Center, Beijing Institute of Brain Disorders Beijing China; ^5^ Department of Neurosurgery Chinese Institute for Brain Research Beijing China

**Keywords:** ascending reticular activating system, disorders of consciousness, intrinsic connectivity networks, the mesocircuit hypothesis

## Abstract

**Background:**

Disorders of consciousness (DoC) lack an integrated framework to guide neuromodulation. This review updates three hierarchical frameworks—ARAS, mesocircuit, and ICNs—and maps therapeutic targets within each.

**Methods:**

We synthesized recent neuroanatomical, optogenetic, neuroimaging, and clinical trial evidence, reevaluating classic frameworks and proposing a hierarchical integration to inform multi‐target strategies.

**Results:**

Three major updates are identified. In ARAS, glutamatergic neurons in PPN/LDT and parabrachial nucleus, rather than cholinergic neurons, are the primary arousal drivers; clinical translation relies on peripheral nerve stimulations with variable efficacy. The mesocircuit is expanded from a unidirectional GPi‐mediated pathway to a bidirectional GPi–GPe balance modulated by dopamine and adenosine; DBS targeting CM‐Pf/CL shows ~44.7% response rate (38/85) but lacks RCTs, whereas amantadine remains the sole Level 1 evidence. Within ICNs, the executive control network has the strongest RCT support, though efficacy depends on etiology and consciousness level; salience network stimulation is untested. These frameworks are hierarchically integrated: ARAS sustains wakefulness, mesocircuit enables cortical activation, and ICNs support conscious content.

**Conclusion:**

Future strategies should adopt multi‐target, multi‐level approaches grounded in individualized network profiling. Priorities include large‐scale RCTs for DBS and stratified protocols based on etiology and consciousness level.

## Graphical abstract

This review integrates three hierarchical frameworks—ascending reticular activating system (ARAS), mesocircuit hypothesis, and intrinsic connectivity networks (ICNs)—to update the neural mechanisms of disorders of consciousness (DoC). We highlight emerging evidence for glutamatergic ARAS pathways, a bidirectional GPi–GPe mesocircuit regulating thalamocortical output, and network‐level disruptions within ICNs. These frameworks jointly inform multi‐level neuromodulation strategies for DoC. Created in BioRender. Zhou, Y. (2026) https://BioRender.com/fuxz6xn.

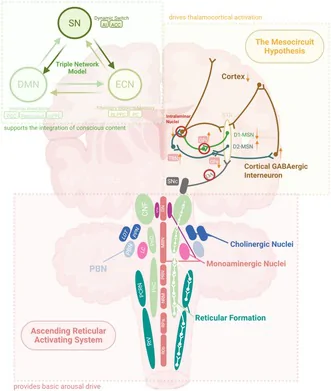

## 1 Introduction

Disorders of consciousness (DoC) represent a pathological condition marked by significant impairment in both the level and content of awareness, resulting from traumatic or non‐traumatic brain injury. According to illness duration, DoC are categorized as acute or prolonged, with prolonged cases typically defined by persistent impairment lasting more than 28 days [1, 2]. Prolonged DoC (pDoC) can be further classified into two primary clinical phenotypes, which are vegetative state/unresponsive wakefulness syndrome (VS/UWS) and minimally conscious state (MCS) [3, 4, 5, 6].

Chronic DoC, commonly arising from various brain injuries such as severe traumatic brain injury, hypoxic encephalopathy, and stroke [7], is characterized by a prolonged clinical course and difficult recovery, severely impacting patients' quality of life while imposing heavy burdens on families and society [8, 9]. In recent years, with the proposal and refinement of theoretical frameworks of consciousness, several potential neuromodulation targets have emerged, and existing neuromodulation studies have attempted interventions targeting different levels [1, 10], offering new possibilities for the treatment of DoC.

However, current stimulation targets are highly heterogeneous, spanning the brainstem, thalamus, and cortex, reflecting a lack of consensus on the neural basis of consciousness. Moreover, existing reviews are often confined to a single framework or technique. Therefore, this review aims to: elaborate and update the ARAS, mesocircuit, and ICN frameworks based on current advances; and systematically review existing and potential neuromodulation targets within each.

## 2 Ascending Reticular Activating System

### 2.1 Definition and Anatomical Boundaries

ARAS is a functional concept describing ascending regulatory systems that maintain arousal, facilitate sleep–wake transitions, and support the arousal component necessary for consciousness [11]. Its anatomical boundaries continue to be updated as technology advances.

**FIGURE 1:**
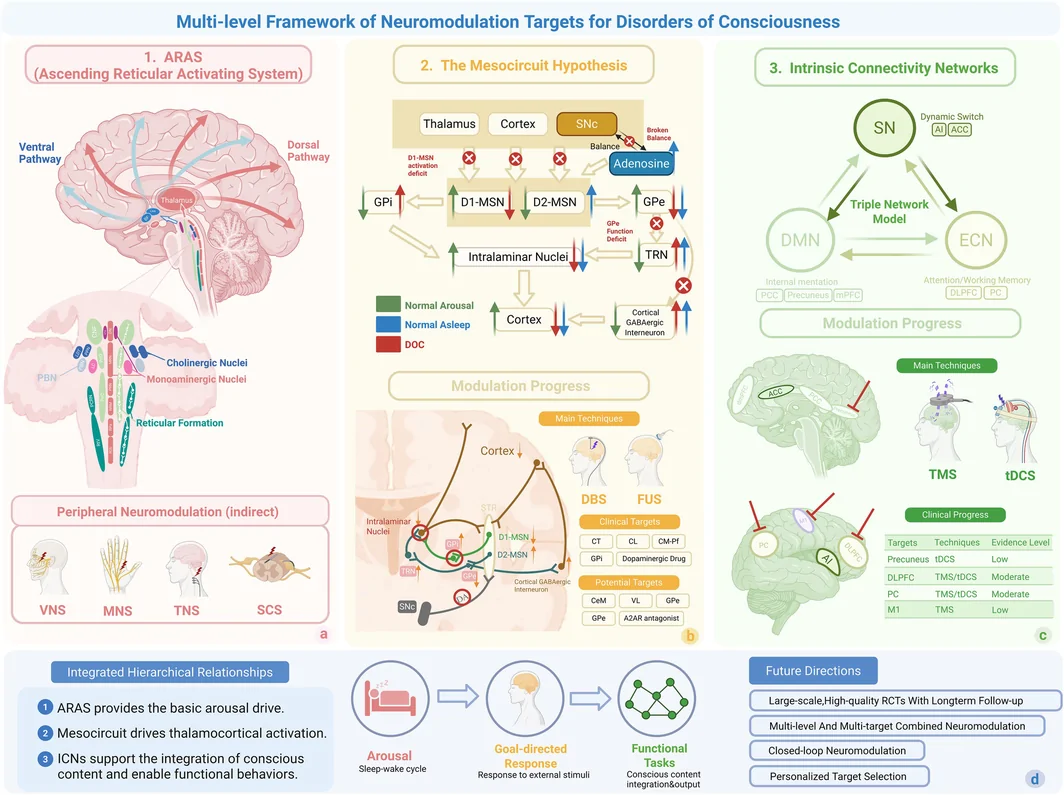
Multi‐level framework of neuromodulation targets for disorders of consciousness. (a) ARAS comprises brainstem reticular formation and key neuromodulatory nuclei, including cholinergic (PPN/LDT), monoaminergic (LC, DRN, TMN, VTA) nuclei and the PBN, which collectively provide the fundamental arousal drive. Peripheral neuromodulation may indirectly modulate ARAS function. (b) The mesocircuit hypothesis describes a cortico–striato–pallido–thalamic loop regulating wake–sleep balance via GPi/GPe and striatal MSNs. Dopamine facilitates D1‐MSN‐mediated thalamocortical disinhibition, whereas adenosine‐driven D2‐MSN activation engages the indirect pathway to suppress cortical activity. Key neuromodulation targets include DBS and FUS of CT, CM‐Pf, CL, and GPi. (c) The intrinsic connectivity networks (ICNs), organized under the triple‐network model (DMN–SN–ECN), support conscious content and network integration. Non‐invasive stimulation targets include the precuneus, DLPFC, posterior parietal cortex (PC/PPC), and M1, primarily using TMS and tDCS. (d) These three frameworks form a hierarchical organization: ARAS supports arousal, the mesocircuit enables thalamocortical activation, and ICNs mediate higher‐order integration. Future neuromodulation should move toward individualized, multi‐target, and network‐based strategies. ARAS, ascending reticular activating system; CL, central lateral nucleus; CM‐Pf, centromedian‐parafascicular complex; CT, central thalamus; D1‐MSN, D1 receptor‐expressing medium spiny neurons; D2‐MSN, D2 receptor‐expressing medium spiny neurons; DBS, deep brain stimulation; DLPFC, dorsolateral prefrontal cortex; DMN, default mode network; DRN, dorsal raphe nucleus; ECN, executive control network; FUS, focused ultrasound stimulation; GPe, globus pallidus externus; GPi, globus pallidus internus; ICNs, intrinsic connectivity networks; LC, locus coeruleus; LDT, laterodorsal tegmental nucleus; M1, primary motor cortex; MNS, median nerve stimulation; MSNs, medium spiny neurons; PBN, parabrachial nucleus; PC/PPC, (posterior) parietal cortex; PPN, pedunculopontine nucleus; SCS, spinal cord stimulation; SN, salience network; SNc, substantia nigra pars compacta; tDCS, transcranial direct current stimulation; TMN, tuberomammillary nucleus; TMS, transcranial magnetic stimulation; TNS, trigeminal nerve stimulation; TRN, thalamic reticular nucleus; VNS, vagus nerve stimulation; VTA, ventral tegmental area. Created in BioRender. Zhou, Y. (2026) https://BioRender.com/odh87in.

As early as the 1940s–1950s, the ARAS was considered synonymous with the brainstem reticular formation itself [11], including the lateral column receiving sensory collaterals and the medial column giving rise to ascending fibers [12]. By the late 20th century, fluorescence histochemistry and chemo‐specificity studies revealed that chemospecific nuclei (cholinergic and monoaminergic) scattered within and outside the reticular formation are core drivers of ARAS. The former project mainly via the dorsal pathway to the intralaminar thalamus, while the latter project mainly via the ventral pathway to the hypothalamus/basal forebrain, thereby inducing cortical activation [13]. In the early 21st century, supplemented by retrograde labeling and optogenetic evidence, thalamic lesions were found to have limited effects on arousal, whereas glutamatergic neurons in the parabrachial nucleus and the adjacent pre‐locus coeruleus area projecting to the basal forebrain are core to arousal [14] (Figure 1a).

Thus, although there is currently no unified, universally accepted anatomical boundary, brainstem reticular formation, the parabrachial nucleus (PBN), the locus coeruleus (LC), the tuberomammillary nucleus (TMN), the ventral tegmental area (VTA), the pedunculopontine nucleus (PPN)/laterodorsal tegmental nucleus (LDT), and the dorsal raphe nucleus (DRN) have all been considered key nodes involved in arousal maintenance and constituents of the ARAS. Therefore, we will now elaborate on the latest research progress and clinical translation of these targets in the field of DOC.

### 2.2 PPN/LDT

The PPN/LDT are known as “cholinergic chemo‐specific nuclei” due to their high proportion of cholinergic neurons [15]. Early electrophysiology combined with histochemical staining showed that PPN/LDT neurons fire at high frequency during wakefulness and REM sleep, with firing locations overlapping cholinergic neuron staining, leading to the classic view that PPN/LDT promote wakefulness via cholinergic mechanisms [16]. However, recent optogenetic and chemogenetic studies reveal that specific activation of PPN/LDT cholinergic neurons mainly promotes REM sleep, with limited effects on wakefulness [17]. In contrast, activation of glutamatergic neurons induces sustained wakefulness [18], suggesting they may be the main drivers of PPN/LDT‐mediated arousal. To date, these studies have used only normal (not DOC) animal models. Notably, bilateral DBS targeting the PPN has only been attempted for refractory epilepsy [19], which provides some inspiration for treatment of DOC.

### 2.3 DRN

The DRN, with its high proportion of 5‐HT neurons, is an important component of ARAS, whose activity is closely related to sleep–wake states supported by electrophysiological evidence [20]. Optogenetic studies indicate that high‐frequency (20–25 Hz) DRN activation directly promotes wakefulness [21, 22, 23], whereas tonic low‐frequency (3–5 Hz) activity indirectly promotes subsequent sleep by accumulating sleep pressure [22]. However, causal evidence for its arousal‐promoting effect comes mainly from normal/chronic pain models, with only one anesthetized mouse study [23]. Whether anesthesia models can recapitulate the condition of DOC patients remains unknown.

### 2.4 LC

The LC is the main source of norepinephrine (NA) in the whole brain. Electrophysiological studies [24] and optogenetic studies [25] have both confirmed that LC activity is closely related to arousal levels and that activating the LC drives the transition from sleep to wakefulness. Moreover, in the field of pharmacology, local administration of the α1‐adrenergic receptor agonist methoxamine into the LC promotes wakefulness [26]. However, to date, the above explorations have been based on normal models rather than DOC models, and there is no clinical neuromodulation or drug therapy targeting this site.

### 2.5 TMN

The TMN is the sole source of the central histaminergic system in the brain [27]. Although electrophysiological monitoring shows that the TMN maintains a high level of tonic discharge throughout wakefulness, providing the baseline tone for initiating and maintaining arousal [28], recent chemogenetic results indicate that activating the TMN does not increase baseline wakefulness [29]. Moreover, there is some disagreement about whether inhibiting the TMN affects NREM sleep [29, 30, 31]. Notably, despite the unclear mechanism, the histamine H3 receptor inverse agonist pitolisant has been approved by the FDA for the treatment of narcolepsy [32], which provides some inspiration for drug treatment of DOC.

### 2.6 VTA

The VTA is a key source of midbrain dopaminergic input. Optogenetic activation (25 Hz) rapidly switches NREM/REM sleep to wakefulness and arouses mice from general anesthesia [33, 34], while VTA dopaminergic neuron inhibition promotes sleep even under strong stimuli [33]. Although no VTA‐targeted neuromodulation exists for DOC, several dopaminergic drugs, including levodopa [35, 36], bromocriptine [37], amantadine [38], and modafinil [39] have entered clinical studies for DOC. However, systemic administration lacks VTA specificity and also affects other dopaminergic areas like the substantia nigra pars compacta (SNc).

### 2.7 PBN

The PBN, located in the rostrodorsal pons, consists mainly of glutamatergic neurons. In normal sleep models, chemogenetic activation of PBN neurons induces sustained wakefulness without sleep rebound, while optogenetic activation triggers immediate arousal that rapidly reverses upon stimulation offset [40]. Besides, chemogenetic activation of PBN neurons shortens the recovery time from anesthesia in rats [41]. Regarding its ascending pathways, although the PBN projects to both the dorsal pathway and the ventral pathway, only activation of the dorsal pathway induces arousal [40]. To date, no neuromodulation or drug therapy targeting the PBN for DOC has been reported.

### 2.8 Summary and Clinical Progress

Taken together, due to the anatomical location of these ARAS nodes around brainstem vital centers, surgical difficulty and risk are high. Therefore, whether now or in the future, the cost‐effectiveness of ARAS as a DBS target is quite low, although some studies have used DBS targeting the mesencephalic reticular formation [42].

Notably, clinical trials of spinal cord stimulation (SCS), vagus nerve stimulation (VNS), median nerve stimulation (MNS), and trigeminal nerve stimulation (TNS) are increasing and have yielded some positive results. However, unlike DBS, none of these techniques have a well‐defined target, and their underlying arousal mechanisms remain inconclusive. Therefore, for rigor, this review does not present them as targeted neuromodulation approaches in the preceding section on brain targets. Instead, their highest‐level evidence is briefly summarized below.

In the SCS field, as a recent non‐randomized controlled cohort study reported, the short‐term SCS group had significantly greater improvement in CRS‐R scores at 3‐month follow‐up, with 49% of patients showing symptom improvement [43]. In the VNS field, one Randomized Controlled Trial (RCT) showed no significant difference in CRS‐R scores between groups before and after tVNS, failing to reach positive conclusions [44]. In the MNS field, one RCT showed that right MNS significantly improved the rate of consciousness recovery at 6 months post‐injury and improved functional outcomes compared with the control group [45]. In the TNS field, one RCT showed that TNS promotes consciousness recovery in patients with pDoC by enhancing regional cerebral metabolism [46].

Finally, from a pharmacological perspective, the downstream receptors of monoaminergic neurotransmitters are mainly G‐protein‐coupled receptors, whereas nicotinic cholinergic receptors and ionotropic glutamate receptors are ion channels. In comparison, the former have slower onset and longer duration, thus mainly exerting modulatory effects. Therefore, for arousal‐promoting drugs, targeting the latter may more easily achieve rapid arousal effects.

## 3 The Mesocircuit Hypothesis

### 3.1 Classical Definition and Anatomical Boundaries

The mesocircuit hypothesis proposed by Schiff centers on the prefrontal cortex–striatum–globus pallidus–thalamus–cortex loop [47]. DoC arises from diffuse brain injury that damages cortico‐striatal and thalamo‐striatal excitatory projections. Loss of these inputs, together with reduced dopaminergic modulation, prevents striatal medium spiny neurons (MSNs) from reaching activation threshold. This enhances internal globus pallidus (GPi) inhibition of the thalamus, further weakening thalamocortical projections and creating a vicious cycle. Although the model still requires refinement regarding the role of the external globus pallidus (GPe) and differential functions of thalamic nuclei, its basic framework accommodates new evidence—notably the involvement of intralaminar thalamic nuclei in arousal.

Intralaminar thalamic nuclei comprise the anterior group and the posterior group with distinct projection patterns. The former includes the centrolateral nucleus (CL), the paracentral nucleus (Pc), and the central medial nucleus (CeM). The latter includes the centromedian nucleus (CM) and the parafascicular nucleus (Pf). Parvalbumin (PV)‐positive CM nuclei project specifically to the striatum, while calbindin (CB)‐positive Pf and anterior intralaminar nuclei project diffusely to prefrontal cortex, striatum, and other areas [48]. Together, they ensure dual driving of striatal MSNs by thalamus and cortex. Using CM and Pf as examples: CM medial part mainly targets the striatal sensorimotor region, while Pf subregions project dispersedly to limbic and association areas [49]. At the output end, the sensorimotor region of GPi projects back to CM, whereas the associative and limbic regions of GPi project to Pf [50]. This closes the positive feedback loop mentioned above.

### 3.2 Revised Mesocircuit Hypothesis

Inspired by motor direct/indirect pathways, the modulatory role of GPe in the mesocircuit has been further clarified. Evidence shows that GPe promotes sleep through two independent pathways that suppress cortical activity, without simply replicating the motor indirect pathway [51]. On the one hand, about the cortex–striatum–GPe–cortex pathway, GABAergic neurons in GPe project directly to the cortex, especially PV‐positive neurons in the anterior GPe [52, 53]. On the other hand, about the cortex–striatum–GPe–thalamic reticular nucleus (TRN)–thalamus–cortex pathway, GPe projects to TRN, indirectly modulating thalamocortical information flow [54, 55].

In healthy individuals, the switching between the GPi and GPe pathways is regulated by dopamine and adenosine. Regarding arousal promotion, dopamine plays a dominant role by, on one hand, enhancing D1‐MSN excitability to facilitate the GPi‐mediated “direct pathway” and promote cortical activation; on the other hand, inhibiting D2‐MSN excitability, which leads to abnormal activation of the GPe, subsequently reducing the activity of STN and cortical inhibitory neurons, thereby facilitating thalamic and cortical activation. When prolonged wakefulness leads to the accumulation of extracellular adenosine to a sufficient concentration [56, 57], adenosine binds to A2A receptors on D2‐MSNs, enhances D2‐MSN excitability, activates the indirect pathway, and ultimately promotes sleep [57].

In patient populations, common causes of disorders of consciousness due to lesions at the mesocircuit level include insufficient excitatory input to D1‐MSNs, GPe atrophy or dysfunction of GPe projections to cortical GABAergic neurons/STN projections [51], as well as an imbalance in the adenosine (A2A) and dopamine (D2) systems (Figure 1b).

### 3.3 Clinical Translation and Potential Therapeutic Targets

#### 3.3.1 Thalamic Nuclei

Early DBS research often targeted the central thalamus (CT), a broad term covering the anterior and posterior intralaminar nuclei, mediodorsal nucleus, and parts of the ventral nuclear group [48]. However, CT‐level electrode placement tends to co‐activate functionally antagonistic nuclei, causing non‐specific interference, while precise localization under 3T MRI is difficult, leading to high targeting errors. Consequently, from the 1990s, Japanese groups led by Tsubokawa and Yamamoto pioneered precise targeting of the posterior intralaminar CM‐Pf complex [58]. Today, CM‐Pf is the most common target for DOC‐DBS. Pooling nine studies from 1990 to date, including 85 patients, the overall response rate is approximately 44.7% (38/85). Nevertheless, the trial design of the highest evidence level for this target is non‐randomized prospective cohort study (Table 1), and the lack of large‐scale RCTs remains the core bottleneck limiting DBS adoption.

**TABLE 1:** Overview of clinical targets related to the Mesocircuit Model and the Intrinsic Connectivity Networks.

Target	Unilateral/Bilateral	Technique	Analyzed number	Diagnosis	Follow‐up time	Eifficacy	OCEBM	GRADE	Citation
CT	Bilateral	DBS	1	1MCS	6 months	CRS‐R Arousal subscale score increase (1/1)	4	Low	[59]
CT	Bilateral	DBS	3	2 VS/UWS,1 MCS	18‐60 months	CRS‐R increase (3/3)	4	Very low	[60]
CT	Bilateral	DBS	1	1MCS	8.5 years	CRS‐R without significant increase (1/1)	4	Very low	[61]
CT	Unilateral L/R	tFUS	10	8MCS, 2VS	1 week	Overall CRS‐R significant increase	4	Very low	[62]
CT	Bilateral	DBS	8	5 VS,3MCS	6 months	CRS‐R increase (3/8)	4	Very low	[63]
CM‐Pf MRF	/	DBS	6CM‐Pf,2MRF	8VS	≥ 6–8 months	PCS increase ≥ 5 (4/8)	4	Very low	[58]
CM‐Pf	Unilateral	DBS	5	5 MCS	≥ 10 years	Achieved communication and mobility out of bed (4/5)	4	Very low	[42]
CM‐Pf	Unilateral	DBS	14	10VS,4MCS	38–60 months	C/NC improvement (5/14)	4	Very low	[64]
CM‐Pf	Bilateral	DBS	1	1MCS	24 months	CRS‐R without significant increase (1/1)	4	Very low	[65]
CM‐Pf	Bilateral	DBS	9	9MCS	6 months	CRS‐R increase (3/9)	2b	Low	[66]
CM‐Pf	Bilateral	DBS	37	13MCS24 VS/UWS	1 year	CRS‐R increase ≥ 3 (12/37)	3	Low	[67]
CM‐Pf	Unilateral R	DBS	4	3 VS, 1MCS	1 months	CRS‐R increase ≥ 1 (4/4)	4	Very low	[68]
CM‐Pf	Bilateral	DBS	9	8 VS, 1MCS	1 months	CRS‐R increase ≥ 1 (3/9)	4	Very low	[68]
CL/DTTm	Bilateral	DBS	5	Cognitive Impairment	90 days	TMT‐B increase ≥ 10% (5/5)	4	Low	[69]
GPi VA	GPi(right) VA(left)	DBS	1	1 Apallic syndrome	19 days	Behavioral and EEG improvement (1/1)	4	Very low	[70]
CT GPi	Bilateral	DBS	5	1UWS,4MCS	11 months	CRS‐R increase (2/5)	4	Very Low	[71]
Precuneus	Midline	HD‐tDCS	11	5VS,6MCS	14 days	CRS‐R increase (9/11)	4	Very Low	[72]
DLPFC	Unilateral R	rTMS	15	15VS	20 days	CRS‐R increase (15/15)	3	Low	[73]
DLPFC	Unilateral R	rTMS	1	1VS	10 months	CRS‐R increase (1/1)	4	Very low	[74]
DLPFC	Unilateral L	rTMS	16	5MCS,11 VS	20 days	CRS‐R increase (9/16)	4	Very low	[75]
DLPFC	Unilateral L	rTMS	20	20VS	4 weeks	CRS‐R increase ≥ 3 (10/20)	2b	Moderate	[76]
DLPFC	Unilateral L	rTMS	25	Mixed VS/MCS	6 weeks	BAEP rank increase (11/25)	2b	Moderate	[77]
DLPFC	Unilateral	TMS	24	24VS	8 weeks	CRS‐R increase	2b	Low	[78]
DLPFC	Unilateral L	tDCS	55	30MCS,25 VS	Immediate assessment	CRS‐R increase (15/55)	1b	Moderate	[79]
DLPFC	Unilateral L	tDCS	16	16MCS	12 days	CRS‐R increase (9/16)	2b	Moderate	[80]
DLPFC	Unilateral L	tDCS	33	17MCS,16VS	3 months	No CRS‐R increase	1b	Low	[81]
PC	Unilateral L	TMS	38	16VS,22 MCS	16 days	CRS‐R increase (19/38)	4	Very low	[82]
PC	Bilateral	tDCS, TMS	1	1MCS	1 month	CRS‐R increase (1/1)	4	Very low	[83]
PC	Midline	tDCS	33	33MCS	10 days	CRS‐R increase (9/33)	1b	Moderate	[84]
PC	Unilateral L	TMS	20	20VS	10 days	CRS‐R increase (8/20)	1b	Moderate	[85]
M1	Unilateral L	TMS	6	3 VS,2 MCS,1eMCS	1 week	CRS‐R increase (1/6)	2b	Low	[86]
M1	Unilateral L	TMS	1	1MCS	6 months	CRS‐R increase (1/1)	4	Very low	[87]
M1	Unilateral L	TMS	11	11VS	1 month	CRS‐R no significant improvement	2b	Low	[88]
M1	Unilateral L	TMS	7	5 MCS,2 VS	2 weeks	CRS‐R increase (1/7)	2b	Low	[89]
M1	Unilateral L	rTMS	33	33VS	4 weeks	CRS‐R increase	2b	Moderate	[90]

*Note:* OCEBM levels: 1b, individual randomized controlled trial (RCT); 2b, low‐quality or small‐sample RCT/non‐randomized controlled cohort study; 3, non‐randomized controlled cohort study; 4, case series or single‐case study. GRADE quality: Low, further research likely to change estimate; Very low, estimate very uncertain; Moderate, further research may change estimate.

Abbreviations: AG, angular gyrus; BAEP, brainstem auditory evoked potential; Bilat, bilateral; C/NC, Coma/Near‐Coma Scale; CL, central lateral nucleus; CM‐Pf, centromedian‐parafascicular complex; CRS‐R, JFK Coma Recovery Scale‐Revised; CT, central thalamus; DBS, deep brain stimulation; DLPFC, dorsolateral prefrontal cortex; DOC, disorders of consciousness; DTTm, medial dorsal tegmental tract; EEG, electroencephalography; FC, functional connectivity; GCS, Glasgow Coma Scale; GPi, globus pallidus interna; GRADE, Grading of Recommendations Assessment, Development and Evaluation; HD‐tDCS, high‐definition transcranial direct current stimulation; IPL, inferior parietal lobe; M1, primary motor cortex; MCS, minimally conscious state; MRF, mesencephalic reticular formation; OCEBM, Oxford Centre for Evidence‐Based Medicine levels of evidence; PC, parietal cortex; PCS, Prolonged Coma Scale (Nihon University); PPC, posterior parietal cortex; rTMS, repetitive transcranial magnetic stimulation; SEP, somatosensory evoked potential; tDCS, transcranial direct current stimulation; tFUS, transcranial focused ultrasound; TMT‐B, Trail Making Test Part B; Unilat., unilateral; UWS, unresponsive wakefulness syndrome; VA, ventral anterior nucleus of thalamus; VS, vegetative state.

Regarding the anterior intralaminar nuclei, Schiff conducted a randomized feasibility study in five patients with preserved consciousness but cognitive impairment, targeting the bilateral CL and the medial dorsal tegmental tract (DTTm) [69]. Processing speed on the Trail Making Test (TMT‐B) improved by > 10% from baseline, but whether these findings generalize to typical DOC populations requires further validation.

To determine the optimal intralaminar target, a single‐center, double‐blind, crossover study (NCT07228286) is currently underway, aiming to compare the CLwith the CM‐Pf, with respect to their relative efficacy in promoting recovery of consciousness. Additionally, targeting nuclei such as CeM and VL has demonstrated arousal‐promoting effects in animal studies [91], but these findings await validation in human studies.

#### 3.3.2 Globus Pallidus (GP)

GP has accumulated robust safety data in the field of movement disorders while in the field of DoC, GPi targeted DBS is still at a very early exploratory stage (Table 1). Hassler first attempted simultaneous stimulation of the right basal pallidum and left lateral polar thalamic nucleus, achieving transient consciousness improvement that faded after 19 days [70]. Lemaire once conducted bilateral thalamic and pallidal stimulation to five patients, showing CRS‐R improvements [71]. However, in the trials mentioned above, the respective contributions of thalamus and pallidum could not be isolated, and the case‐series design limits generalizability.

As for the GPe, due to its anatomical adjacency to the GPi, current spread makes functional separation difficult. Moreover, the GPe plays an irreplaceable role in the indirect motor pathway, and its isolated inhibition may induce motor side effects. To date, no systematic animal or human studies are available for reference.

#### 3.3.3 Pharmacological Modulation

PET studies have shown significant dopamine depletion at dopaminergic presynaptic terminals in some post‐TBI patients with MCS, with the degree of depletion negatively correlated with CRS‐R scores, providing a neurobiological rationale for dopamine‐targeted interventions [92]. Both bromocriptine and levodopa have been investigated in clinical studies. Bromocriptine may promote arousal by activating D2‐MSNs to attenuate GPe‐mediated indirect pathway activity, but evidence is limited to case reports and very small samples [93]. Levodopa may directly replenish dopamine deficiency in the SNc‐striatal pathway, yet has only been explored in small open‐label trials [94]. To date, the only pharmacological intervention for DOC supported by Level 1 evidence is the indirect dopaminergic agonist amantadine [38], which has been included as a Level B recommendation for adult post‐traumatic DOC in the 2018 DOC practice guidelines [95]. However, the evidence base is restricted to traumatic etiology, and its applicability to non‐traumatic patients awaits dedicated validation.

Overall, the existing evidence base for DBS interventions in DOC remains low, with the highest trial design reaching only OCEBM Level 2, and GRADE ratings mostly falling within the Low to Very Low range. Consequently, DBS studies urgently require standardized patient inclusion criteria, a unified efficacy evaluation system, and refined subgroup analyses. In the realm of pharmacological modulation, patient stratification using dopamine transporter–single‐photon emission computed tomography (DAT‐SPECT) prior to enrollment may improve the response rate to dopaminergic agents [92]. Meanwhile, continued exploration of novel targeted drugs, such as adenosine A2A receptor antagonists, is warranted.

## 4 Intrinsic Connectivity Networks (ICNs)

### 4.1 Definition of ICNs and the Triple Network Model

Intrinsic connectivity networks (ICNs) are large‐scale brain networks that exhibit stable functional connectivity during rest and remain identifiable during task states, including the default mode network (DMN), salience network (SN), executive control network (ECN), dorsal or ventral attention network, and sensorimotor network (SMN) and so on [96]. In the field of disorders of consciousness research, the Triple Network Model integrates the DMN, SN, and ECN into a unified framework, posing that SN serves as a dynamic switching hub, coordinating the processing of internal and external information by regulating the balance of activation between the ECN and DMN [97] and positing that functional disconnection among these three networks is the core basis of the neurophysiological mechanisms underlying DOC [98, 99] (Figure 1c).

### 4.2 Default Mode Network

DMN is anchored by the posterior cingulate cortex (PCC)/precuneus and medial prefrontal cortex (mPFC) as its core hubs, along with the dorsal medial prefrontal subsystem (dMPFC) and medial temporal lobe subsystem (MTL), which primarily supports internal cognitions such as memory, emotion, and self‐referential thinking [100, 101]. fMRI findings have shown that DMN internal connectivity strength [102] and the connectivity with anticorrelated networks [103] are significantly positively correlated with the level of consciousness, providing a theoretical basis for neuromodulation interventions targeting core DMN nodes. One study applied transcranial direct current stimulation(tDCS) targeting the precuneus in 11 patients with pDOC and the pre‐post self‐controlled results showed that CRS‐R scores improved in 9 patients [72]. However, further higher‐quality studies are needed to confirm its efficacy.

### 4.3 Salience Network

SN's core nodes lie in the anterior insula (AI) and dorsal anterior cingulate cortex (dACC). fMRI analyses have revealed that patients in VS show a marked reduction in SN internal synchrony and spontaneous neural activity compared to healthy controls [104], which suggests that the SN may be a potential target. However, to date, no neuromodulation therapy targeting this network has been reported.

### 4.4 Executive Control Network

ECN is anchored by the dorsolateral prefrontal cortex (DLPFC) and the posterior parietal cortex as its core nodes. It subserves higher‐order functions such as sustained attention, working memory, and complex cognitive integration [96]. In patients with DoC, this network typically exhibits a marked reduction in functional connectivity [105], making it an important target for neuromodulation. Numerous non‐invasive brain stimulation studies targeting the DLPFC and posterior parietal cortex have accumulated to date and specific data are summarized as follows:

As shown in Table 1, the most abundant studies target the DLPFC, encompassing both repetitive transcranial magnetic stimulation (rTMS) and tDCS. Regarding TMS, two moderate‐quality RCTs targeting the left DLPFC each demonstrated varying degrees of CRS‐R improvement [76, 77]. In addition, two studies targeting the right DLPFC (引) also reported CRS‐R improvement [74, 75], but these were single‐center, small‐sample, or case studies with low levels of evidence.

Regarding tDCS, two moderate‐quality RCTs by Thibaut and Martial established initial evidence for left DLPFC tDCS, both showing significant CRS‐R improvement [79, 80]. However, a multicenter sham‐controlled RCT was terminated early due to no overall group difference [81]. Post hoc subgroup analysis revealed marked improvement at 3 months in MCS and traumatic etiology patients, suggesting efficacy depends heavily on etiology and consciousness level.

Research on posterior parietal cortex (PC) targets is also well documented. One tDCS study targeting the midline parietal cortex [84] and one TMS study targeting the left parietal cortex each showed moderate CRS‐R improvement [85].

### 4.5 Sensorimotor Network

SMN is anchored by the primary motor cortex (M1), primary somatosensory cortex (S1), premotor cortex (PMC), and supplementary motor area (SMA) as its core nodes. Through the cortico‐basal ganglia‐thalamo‐cortical loop and the corticospinal tract, it coordinates sensory integration and motor output [96]. Notably, the modulatory effects of M1 are not confined to the sensorimotor loop itself—M1 stimulation can retrograde‐activate thalamocortical pathways to increase global cortical excitability, thereby providing a mechanistic basis for “bottom‐up” arousal promotion [89, 106]. This constitutes the core theoretical rationale for targeting M1 in DoC neuromodulation.

All existing clinical studies targeting M1 have employed rTMS, predominantly using a 20 Hz high‐frequency protocol. However, its clinical efficacy remains questionable (Table 1). A crossover randomized controlled trial [88], a sham‐controlled study [89], and a RCT [86] have all shown limited CRS‐R improvement. Meanwhile, meta‐analyses have evaluated and compared the effect sizes of rTMS targeting M1 versus the DLPFC [107]. The results indicate that, although the effect size for CRS‐R improvement with M1 targeting is significant, it is substantially lower than that for DLPFC targeting. In frequency subgroup analyses, only 20 Hz rTMS showed statistically significant effects, whereas the 10 Hz rTMS group did not exhibit significant changes.

The main limitation of current research is that most studies treat each network node as an independent intervention target, whereas consciousness emergence and maintenance rely on dynamic triple‐network balance, thus it's quite difficult for an isolated activation of a single node to systematically restore the global coupling. Future studies should therefore develop cross‐network, synergistic stimulation protocols based on the triple‐network model. Furthermore, high patient‐level heterogeneity must be addressed which means stratified, multicenter, large‐sample RCTs are needed to obtain clinically meaningful target‐population matching evidence. Last but not least, given the heterogeneity in network disruption patterns across DoC patients, an individualized network mapping framework for precise target selection should also be established.

## 5 Conclusion

To address the fragmented and outdated understanding of the neural basis of disorders of consciousness in clinical practice, this review systematically updates three theoretical frameworks—ARAS, the Mesocircuit Hypothesis, and ICNs—and maps existing and potential neuromodulatory targets within each.

ARAS functions as a hierarchically organized arousal system, transmitting ascending signals via dorsal and ventral pathways to achieve cortical activation. A critical theoretical advance is the identification, through optogenetic and chemogenetic approaches, that glutamatergic rather than cholinergic neurons in nuclei such as PPN/LDT serve as the primary drivers of arousal, implicating ionotropic neurotransmitter systems as the principal pharmacological targets. Given the proximity of ARAS nuclei to brainstem vital centers, current interventions rely predominantly on pharmacological agents and indirect peripheral neurostimulation, including SCS, VNS, MNS, TNS, whose mechanisms and precise targets remain incompletely understood.

The updated mesocircuit hypothesis extends beyond a unidirectional GPi‐mediated arousal pathway to a bidirectional arousal–sleep balance co‐regulated by both GPi and GPe, maintained through dynamic dopaminergic and adenosinergic modulation. DBS remains the primary modality at this level, with thalamic targeting progressively refined from the broad “central thalamus” to specific nuclei including CM‐Pf and CL, while direct GPi‐targeted interventions remain scarce. Dopaminergic pharmacotherapy also operates principally at this level.

Among ICNs, neuromodulation of the DMN precuneus remains exploratory, supported only by low‐quality uncontrolled studies. The SN, a critical switching hub in the triple‐network model, lacks any direct stimulation studies targeting the AI or dACC, representing a conspicuous gap between neuroimaging evidence and clinical translation. ECN targets—DLPFC and PPC—have accumulated the strongest and most extensive RCT evidence to date, yet are constrained by small sample sizes, high parameter heterogeneity, and absent long‐term follow‐up data. SMN target M1 has the longest investigative history but remains limited by low and inconsistent evidence, with its principal value possibly residing in bottom‐up thalamocortical arousal facilitation rather than direct restoration of conscious content integration.

Crucially, these three frameworks are not parallel but hierarchically integrated. Intact ARAS function establishes the baseline excitability of prefrontal cortex and thalamus, enabling arousal and sleep–wake cycling. Upon this foundation, medium spiny neurons within the Mesocircuit Hypothesis can breach activation thresholds, disinhibit thalamic output, and drive the broad cortical activation underlying goal‐directed behavior. Only with sustained cortical activation can ICNs operate efficiently, support dynamic inter‐network switching, and enable functional task performance. This hierarchical logic should not only guide mechanistic understanding of DoC but also inform the design of future multi‐target, multi‐level neuromodulation strategies grounded in individualized network injury mapping (Figure 1d).

## Funding

This study was funded by International (Hong Kong, Macao, and Taiwan) Science and Technology Cooperation Project (Z221100002722014), Science and Technology Innovation 2030 (2022ZD0205300), Chinese Institute for Brain Research Youth Scholar Program (2022‐NKX‐XM‐02), National Natural Science Foundation of China (82371197), Natural Science Foundation of Beijing Municipality (7232049) and STI2030‐Major Projects (2021ZD0200200). The funding source had no role in the study design, data collection, analysis, interpretation, or the decision to submit this article for publication. All authors declare no other competing interests.

## Conflicts of Interest

The authors declare no conflicts of interest.

## Data Availability

This manuscript is a narrative review. The data supporting the conclusions of this article are derived from previously published studies, which are cited throughout the text. Any summarized data (e.g., response rates from clinical trials) presented in tables and figures are available from the corresponding author upon reasonable request.
